# Supplementary material for: Comparative Efficacy of Various Exercise Therapies and Combined Treatments on Inflammatory Biomarkers and Morphological Measures of Skeletal Muscle among Older Adults with Knee Osteoarthritis: A Network Meta-Analysis
Source: Biomedicines. 2024 Jul 9;12(7):1524. doi: 10.3390/biomedicines12071524 (PMC11275072; doi:10.3390/biomedicines12071524)

Supplementary table S1

Table S1. Database search formulas

Data base	Search terms for query
**Pubmed**	
#1	((elder adults) OR order adults) OR middle-aged adults
#2	((knee osteoarthritis) OR gonarthritis) OR knee arthritis)
#3	(((exercise training) OR resistance exercise) OR aerobic training) OR physical activity
#4	((((muscle morphology) OR muscle architecture) OR muscle ((thickness) OR cross section area) OR muscle CSA) OR muscle hypertrophy)
#5	((inflammatory markers) OR proinflammatory cytokine) OR C-reactive protein
#6	#4 OR #5
#7	(Randomized controlled trial) OR Randomization
#8	((((#1) AND #3) AND #6) AND #7
**Physiotherapy Evidence Database (PEDro)**	
	Method: clinical trial
	Abstract & Title:
#1	knee osteoarthritis
#2	exercise training
#3	physical activity
#4	muscle cross-sectional area
#5	muscle thickness
#6	inflammatory markers
#7	proinflammatory cytokine
#8	Randomized controlled trial
**China knowledge resource integrated database**	
#1	knee osteoarthritis
#2	exercise training
#3	(muscle cross sectional area) OR (muscle thickness) OR (muscle hypertrophy)
#4	(inflammatory markers) OR (proinflammatory cytokine)
#5	#3 OR #4
#6	randomized controlled trial
#7	#1 AND #2 AND #5 AND #6

(continued)

Table S1. (continued)

Data base	Search terms for query
**Excerpta Medica dataBASE (EMBASE)**	
#1	'knee osteoarthritis'
#2	'exercise training'
#3	'physical activity'
#4	'muscle cross sectional area'
#5	'muscle thickness'
#6	'muscle hypertrophy'
#7	'muscle morphology'
#8	'inflammatory markers'
#9	'proinflammatory cytokine'
#10	#2 OR #3
#11	#4 OR #5 OR #6 OR #7 OR #8 OR #9
#12	#1 AND #10 AND #11 AND ([systematic review]/lim OR [meta analysis]/lim OR [randomized controlled trial]/lim) AND [humans]/lim
**Cochrane Library Database**	
#1	elderly
#2	knee osteoarthritis
#3	exercise training
#4	resistance training
#5	aerobic training
#6	physical activity
#7	proinflammatory cytokine
#8	inflammatory markers
#9	C-reactive protein
#10	muscle thickness
#11	muscle hypertrophy
#12	muscle cross sectional area
#13	#3 OR #4 OR #5 OR #6
#14	#7 OR #8 OR #9 OR #10 OR #11 OR #12
#15	randomized controlled trial
#16	#1 AND #2 AND #13 AND #14 AND #15

(continued)

Table S1. (continued)

Data base	Search terms for query
**Google Scholar**	
#1	allintitle: "elderly" OR "older adults"
#2	allintitle: knee osteoarthritis
#3	allintitle: "exercise training" OR "physical activity"
#4	allintitle: “muscle cross sectional area” OR “muscle thickness” OR “muscle hypertrophy”
#6	allintitle: "inflammatory markers" OR "proinflammatory cytokine"
#5	allintitle: randomized controlled trial

Supplementary Table S2

Table S2. League table for pairwise and network meta-analysis of mean change in muscle volume from baseline.

	Direct evidence of pairwise meta-analyses (row compared with column)																				
	**V1**	**V2**	**V3**	**V4**	**V5**	**V6**	**V7**	**V8**	**V9**	**V10**	**V11**	**V12**	**V13**	**V14**	**V15**	**V16**	**V17**	**V18**	**V19**	**V20**	**V21**
**V1**	AET + BFR	.	.	.	.	.	.	.	.	.	0.27 (-0.97; 1.51)	.	.	.	.	.	.	.	.	.	0.50 (-0.74; 1.75)
**V2**	0.31 (-1.19; 1.80)	IKET + PAM	.	-0.08 (-1.15; 0.98)	.	.	0.08 (-0.99; 1.16)	.	.	.	.	.	.	.	.	.	.	.	.	.	0.09 (-0.92; 1.10)
**V3**	-0.34 (-1.79; 1.12)	-0.64 (-1.96; 0.67)	IMET (Active) + BioF	.	.	.	.	.	.	.	.	.	.	.	.	.	.	.	0.40 (-0.67; 1.47)	.	0.70 (-0.47; 1.88)
**V4**	0.08 (-1.29; 1.44)	-0.23 (-1.23; 0.77)	0.41 (-0.75; 1.57)	IMET (Active) + PAM	-0.27 (-1.09; 0.56)	.	0.17 (-0.90; 1.24)	.	.	.	.	.	.	.	.	.	.	.	.	.	0.17 (-0.83; 1.17)
**V5**	-0.37 (-1.69; 0.95)	-0.68 (-1.74; 0.39)	-0.03 (-1.13; 1.07)	-0.45 (-1.14; 0.24)	IMET (NMES) + PAM	.	.	.	.	.	.	.	.	.	.	.	.	.	.	0.14 (-0.67; 0.94)	**1.15 (0.33; 1.97)**
**V6**	-0.77 (-2.09; 0.55)	-1.08 (-2.26; 0.10)	-0.43 (-1.56; 0.70)	-0.85 (-1.86; 0.16)	-0.40 (-1.34; 0.54)	LIRET + BFR	.	.	.	.	.	.	.	-0.06 (-1.18; 1.05)	0.18 (-1.01; 1.36)	**0.98 (0.19; 1.78)**	.	.	.	.	**1.72 (0.87; 2.58)**
**V7**	0.39 (-1.11; 1.89)	0.08 (-0.99; 1.16)	0.73 (-0.59; 2.05)	0.31 (-0.69; 1.32)	0.76 (-0.31; 1.83)	1.16 (-0.02; 2.35)	LIRET + PAM	.	.	.	.	.	.	.	.	.	.	.	.	.	0.00 (-1.01; 1.02)
**V8**	0.43 (-1.04; 1.89)	0.12 (-1.21; 1.45)	0.76 (-0.52; 2.04)	0.35 (-0.83; 1.53)	0.79 (-0.33; 1.92)	**1.20 (0.06; 2.34)**	0.03 (-1.30; 1.37)	MET + ND	.	.	.	.	.	.	.	.	.	0.40 (-0.51; 1.31)	.	.	0.09 (-0.82; 1.00)
**V9**	0.33 (-1.29; 1.95)	0.02 (-1.46; 1.50)	0.67 (-0.76; 2.09)	0.25 (-1.07; 1.57)	0.70 (-0.54; 1.93)	1.10 (-0.22; 2.42)	-0.06 (-1.55; 1.42)	-0.10 (-1.56; 1.37)	MET + PAM	.	.	.	.	.	.	.	.	.	.	-0.56 (-1.58; 0.45)	.
**V10**	-0.05 (-1.40; 1.29)	-0.36 (-1.56; 0.83)	0.28 (-0.86; 1.43)	-0.13 (-1.16; 0.90)	0.32 (-0.65; 1.28)	0.72 (-0.27; 1.70)	-0.44 (-1.64; 0.75)	-0.48 (-1.63; 0.67)	-0.38 (-1.73; 0.97)	MET + TCM	.	.	.	.	.	.	.	.	.	.	0.57 (-0.14; 1.27)
**V11**	0.25 (-0.89; 1.40)	-0.05 (-1.28; 1.18)	0.59 (-0.59; 1.77)	0.18 (-0.89; 1.25)	0.62 (-0.38; 1.63)	**1.02 (0.03; 2.02)**	-0.14 (-1.37; 1.10)	-0.17 (-1.36; 1.02)	-0.07 (-1.45; 1.30)	0.31 (-0.73; 1.35)	AET	.	.	.	-0.47 (-1.54; 0.60)	.	.	.	.	.	0.28 (-0.53; 1.09)
**V12**	0.58 (-1.05; 2.20)	0.27 (-1.24; 1.78)	0.91 (-0.55; 2.38)	0.50 (-0.88; 1.88)	0.94 (-0.39; 2.28)	**1.35 (0.00; 2.69)**	0.18 (-1.33; 1.70)	0.15 (-1.01; 1.31)	0.25 (-1.39; 1.88)	0.63 (-0.73; 1.98)	0.32 (-1.07; 1.71)	AQET	.	.	.	.	.	0.25 (-0.47; 0.96)	.	.	.
**V13**	0.37 (-1.34; 2.09)	0.06 (-1.54; 1.66)	0.71 (-0.85; 2.27)	0.29 (-1.19; 1.77)	0.74 (-0.70; 2.18)	1.14 (-0.31; 2.59)	-0.02 (-1.62; 1.58)	-0.05 (-1.62; 1.51)	0.04 (-1.68; 1.76)	0.42 (-1.03; 1.88)	0.12 (-1.37; 1.61)	-0.20 (-1.93; 1.52)	IKET	.	.	.	.	.	.	.	0.14 (-1.14; 1.42)
**V14**	-0.11 (-1.40; 1.17)	-0.42 (-1.56; 0.72)	0.22 (-0.86; 1.31)	-0.19 (-1.15; 0.77)	0.26 (-0.62; 1.13)	0.66 (-0.07; 1.39)	-0.50 (-1.65; 0.64)	-0.54 (-1.64; 0.56)	-0.44 (-1.71; 0.82)	-0.06 (-1.00; 0.88)	-0.37 (-1.32; 0.58)	-0.69 (-2.00; 0.62)	-0.48 (-1.90; 0.94)	HIRET	0.01 (-0.92; 0.95)	0.36 (-0.75; 1.48)	.	.	.	-0.07 (-1.30; 1.17)	0.23 (-0.53; 1.00)
**V15**	-0.19 (-1.42; 1.05)	-0.50 (-1.62; 0.63)	0.15 (-0.92; 1.21)	-0.27 (-1.20; 0.67)	0.18 (-0.68; 1.05)	0.58 (-0.19; 1.35)	-0.58 (-1.71; 0.55)	-0.61 (-1.69; 0.46)	-0.52 (-1.79; 0.76)	-0.13 (-1.04; 0.77)	-0.44 (-1.28; 0.40)	-0.76 (-2.06; 0.53)	-0.56 (-1.96; 0.84)	-0.07 (-0.78; 0.63)	MIRET	.	.	.	.	.	**0.62 (0.01; 1.23)**
**V16**	0.32 (-1.14; 1.79)	0.02 (-1.33; 1.36)	0.66 (-0.63; 1.96)	0.25 (-0.95; 1.44)	0.69 (-0.44; 1.83)	**1.09 (0.33; 1.86)**	-0.07 (-1.41; 1.28)	-0.10 (-1.41; 1.20)	-0.00 (-1.47; 1.46)	0.38 (-0.80; 1.55)	0.07 (-1.11; 1.25)	-0.25 (-1.74; 1.24)	-0.05 (-1.63; 1.54)	0.44 (-0.47; 1.34)	0.51 (-0.49; 1.51)	LIRET	.	.	.	.	.
**V17**	0.42 (-1.24; 2.07)	0.11 (-1.43; 1.65)	0.75 (-0.74; 2.25)	0.34 (-1.07; 1.75)	0.79 (-0.58; 2.15)	1.19 (-0.19; 2.57)	0.03 (-1.52; 1.57)	-0.01 (-1.51; 1.50)	0.09 (-1.57; 1.75)	0.47 (-0.92; 1.86)	0.16 (-1.26; 1.58)	-0.16 (-1.82; 1.51)	0.05 (-1.70; 1.80)	0.53 (-0.82; 1.88)	0.61 (-0.72; 1.93)	0.09 (-1.43; 1.61)	MBT	.	.	.	0.10 (-1.10; 1.29)
**V18**	0.82 (-0.64; 2.29)	0.52 (-0.81; 1.84)	1.16 (-0.12; 2.44)	0.75 (-0.43; 1.92)	**1.19 (0.07; 2.31)**	**1.59 (0.45; 2.73)**	0.43 (-0.90; 1.76)	0.40 (-0.51; 1.31)	0.49 (-0.97; 1.96)	0.88 (-0.28; 2.03)	0.57 (-0.62; 1.76)	0.25 (-0.47; 0.96)	0.45 (-1.12; 2.02)	0.94 (-0.16; 2.04)	1.01 (-0.07; 2.09)	0.50 (-0.81; 1.81)	0.41 (-1.10; 1.91)	MET	.	.	-0.31 (-1.22; 0.60)
**V19**	-0.06 (-1.45; 1.33)	-0.37 (-1.61; 0.87)	0.28 (-0.59; 1.15)	-0.14 (-1.20; 0.93)	0.31 (-0.68; 1.30)	0.71 (-0.33; 1.75)	-0.45 (-1.69; 0.79)	-0.48 (-1.69; 0.72)	-0.39 (-1.72; 0.95)	-0.01 (-1.06; 1.05)	-0.31 (-1.41; 0.78)	-0.63 (-2.04; 0.77)	-0.43 (-1.93; 1.07)	0.05 (-0.93; 1.04)	0.13 (-0.84; 1.10)	-0.38 (-1.60; 0.84)	-0.48 (-1.91; 0.96)	-0.88 (-2.09; 0.32)	IMET (Active)	-0.90 (-2.34; 0.54)	**1.15 (0.02; 2.27)**
**V20**	-0.23 (-1.50; 1.03)	-0.54 (-1.62; 0.53)	0.10 (-0.90; 1.11)	-0.31 (-1.16; 0.53)	0.13 (-0.56; 0.83)	0.54 (-0.31; 1.38)	-0.63 (-1.71; 0.46)	-0.66 (-1.72; 0.40)	-0.56 (-1.58; 0.45)	-0.18 (-1.07; 0.71)	-0.49 (-1.42; 0.44)	-0.81 (-2.09; 0.47)	-0.61 (-1.99; 0.78)	-0.12 (-0.88; 0.63)	-0.05 (-0.82; 0.72)	-0.56 (-1.61; 0.49)	-0.65 (-1.97; 0.66)	-1.06 (-2.12; 0.00)	-0.18 (-1.04; 0.69)	IMET (NMES)	**0.67 (0.07; 1.27)**
**V21**	0.51 (-0.63; 1.66)	0.21 (-0.76; 1.17)	0.85 (-0.05; 1.75)	0.44 (-0.31; 1.18)	**0.88 (0.22; 1.54)**	**1.28 (0.60; 1.97)**	0.12 (-0.85; 1.09)	0.09 (-0.82; 1.00)	0.18 (-0.97; 1.33)	0.57 (-0.14; 1.27)	0.26 (-0.51; 1.02)	-0.06 (-1.22; 1.10)	0.14 (-1.14; 1.42)	**0.63 (0.01; 1.24)**	**0.70 (0.13; 1.27)**	0.19 (-0.75; 1.13)	0.10 (-1.10; 1.29)	-0.31 (-1.22; 0.60)	0.57 (-0.22; 1.36)	**0.75 (0.21; 1.29)**	RC
	**V1**	**V2**	**V3**	**V4**	**V5**	**V6**	**V7**	**V8**	**V9**	**V10**	**V11**	**V12**	**V13**	**V14**	**V15**	**V16**	**V17**	**V18**	**V19**	**V20**	**V21**
	Relative effects of NMA (column compared with row)																				

Pairwise (upper right portion) and network (lower left portion) meta-analysis results are presented for mean change (from baseline) in pain outcomes. Effect estimation is presented in standardized mean difference (SMD) with 95% CI. Significant results are marked in bold.

NMA, network meta-analysis; AET, aerobic exercise training; AQET, aquatic exercise therapy; BFR, blood flow restriction; BioF, biofeedback; HIRET, high-intensity resistance exercise training; IKET, isokinetic exercise training; IMET, isometric exercise training; LIRET, low-intensity resistance exercise training; MBT, mind-body therapy; MET, multicomponent exercise training; MIRET, medium-intensity resistance exercise training; ND, nutrition and diet; NMES, neuromuscular electrical stimulation; PAM, physical agent modality; RC, regular care; TCM, traditional Chinese medicine.

Supplementary Table S3

Table S3. League table for pairwise and network meta-analysis of mean change in serum level of inflammation from baseline.

	Direct evidence of pairwise meta-analyses (row compared with column)																			
	**V1**	**V2**	**V3**	**V4**	**V5**	**V6**	**V7**	**V8**	**V9**	**V10**	**V11**	**V12**	**V13**	**V14**	**V15**	**V16**	**V17**	**V18**	**V19**	**V20**
**V1**	IKET + PAM	.	.	.	.	.	.	.	.	**-1.98 (-2.83; -1.12)**	.	.	.	.	.	.	.	.	.	**-1.35 (-2.25; -0.46)**
**V2**	-0.34 (-1.40; 0.72)	IKET + TCM	.	.	.	.	.	.	.	**-1.14 (-1.93; -0.35)**	.	.	.	.	.	.	.	.	.	.
**V3**	**-2.70 (-4.06; -1.34)**	**-2.36 (-3.94; -0.78)**	IMET (Active) + ND	.	.	.	.	.	.	.	0.45 (-0.47; 1.37)	.	.	.	.	.	.	.	.	.
**V4**	-0.65 (-1.61; 0.30)	-0.31 (-1.56; 0.94)	**2.05 (0.77; 3.32)**	IMET (Active) + TCM	.	.	.	.	.	.	.	.	.	-0.71 (-1.54; 0.13)	.	.	.	.	.	**-1.61 (-2.45; -0.78)**
**V5**	-1.24 (-2.49; 0.02)	-0.90 (-2.39; 0.60)	**1.46 (0.09; 2.83)**	-0.58 (-1.76; 0.59)	IMET (Active) + WBV	.	.	.	.	.	-0.54 (-1.64; 0.56)	.	.	.	.	.	.	.	.	**-1.14 (-2.25; -0.03)**
**V6**	-1.33 (-2.71; 0.04)	-0.99 (-2.59; 0.60)	1.36 (-0.27; 3.00)	-0.68 (-2.00; 0.64)	-0.10 (-1.65; 1.45)	LIRET + BFR	.	.	.	.	.	.	-0.29 (-1.27; 0.70)	.	.	.	.	.	.	.
**V7**	-1.13 (-2.36; 0.11)	-0.79 (-2.26; 0.69)	**1.57 (0.05; 3.09)**	-0.47 (-1.65; 0.70)	0.11 (-1.32; 1.54)	0.21 (-1.18; 1.60)	MIRET + PAM	.	.	.	.	.	-0.28 (-1.34; 0.78)	.	.	.	.	.	.	-1.04 (-2.16; 0.08)
**V8**	**-1.70 (-2.49; -0.90)**	**-1.36 (-2.49; -0.23)**	1.00 (-0.16; 2.16)	**-1.04 (-1.70; -0.39)**	-0.46 (-1.51; 0.59)	-0.36 (-1.57; 0.84)	-0.57 (-1.62; 0.47)	MET + ND	.	.	.	.	.	-0.13 (-0.50; 0.24)	.	.	.	.	.	-0.12 (-0.45; 0.21)
**V9**	-0.69 (-1.65; 0.26)	-0.35 (-1.60; 0.89)	**2.00 (0.70; 3.31)**	-0.04 (-0.91; 0.83)	0.54 (-0.65; 1.73)	0.64 (-0.68; 1.96)	0.43 (-0.74; 1.60)	**1.00 (0.31; 1.69)**	MET + TCM	.	.	.	.	.	.	.	.	.	.	**-1.19 (-1.81; -0.58)**
**V10**	**-1.48 (-2.20; -0.77)**	**-1.14 (-1.93; -0.35)**	1.22 (-0.15; 2.58)	-0.83 (-1.80; 0.14)	-0.25 (-1.51; 1.02)	-0.15 (-1.53; 1.23)	-0.36 (-1.60; 0.89)	0.21 (-0.60; 1.02)	-0.79 (-1.76; 0.18)	IKET	.	.	.	.	-0.27 (-1.30; 0.76)	.	.	.	.	-0.77 (-1.82; 0.28)
**V11**	**-2.25 (-3.25; -1.24)**	**-1.91 (-3.19; -0.62)**	0.45 (-0.47; 1.37)	**-1.59 (-2.47; -0.72)**	-1.01 (-2.03; 0.01)	-0.91 (-2.27; 0.44)	-1.12 (-2.34; 0.09)	-0.55 (-1.26; 0.16)	**-1.55 (-2.48; -0.63)**	-0.76 (-1.78; 0.25)	IMET (Active)	.	.	**1.01 (0.21; 1.81)**	.	.	.	.	.	-0.60 (-1.70; 0.50)
**V12**	**-1.74 (-2.70; -0.78)**	**-1.40 (-2.65; -0.15)**	0.96 (-0.35; 2.26)	**-1.09 (-1.96; -0.21)**	-0.50 (-1.70; 0.69)	-0.40 (-1.61; 0.80)	-0.61 (-1.72; 0.49)	-0.04 (-0.74; 0.66)	**-1.04 (-1.92; -0.17)**	-0.26 (-1.22; 0.71)	0.51 (-0.42; 1.44)	HIRET	-0.04 (-0.82; 0.74)	.	.	.	0.67 (-0.63; 1.97)	.	.	-0.10 (-0.76; 0.57)
**V13**	**-1.62 (-2.58; -0.67)**	**-1.28 (-2.53; -0.03)**	1.08 (-0.23; 2.38)	**-0.97 (-1.84; -0.10)**	-0.39 (-1.58; 0.81)	-0.29 (-1.27; 0.70)	-0.50 (-1.47; 0.48)	0.07 (-0.62; 0.77)	**-0.93 (-1.80; -0.06)**	-0.14 (-1.11; 0.83)	0.62 (-0.30; 1.55)	0.12 (-0.58; 0.81)	MIRET	.	.	.	.	.	.	-0.29 (-0.92; 0.35)
**V14**	**-1.74 (-2.55; -0.93)**	**-1.40 (-2.54; -0.26)**	0.96 (-0.17; 2.09)	**-1.09 (-1.71; -0.47)**	-0.51 (-1.55; 0.54)	-0.41 (-1.62; 0.81)	-0.62 (-1.68; 0.44)	-0.04 (-0.38; 0.30)	**-1.05 (-1.76; -0.34)**	-0.26 (-1.09; 0.57)	0.51 (-0.15; 1.17)	-0.00 (-0.72; 0.72)	-0.12 (-0.83; 0.60)	MET	.	.	.	.	0.02 (-0.84; 0.88)	0.13 (-0.32; 0.57)
**V15**	**-1.05 (-1.89; -0.21)**	-0.71 (-1.83; 0.42)	**1.65 (0.39; 2.91)**	-0.40 (-1.20; 0.41)	0.19 (-0.96; 1.33)	0.29 (-0.98; 1.56)	0.08 (-1.04; 1.20)	**0.65 (0.05; 1.25)**	-0.35 (-1.16; 0.45)	0.43 (-0.37; 1.24)	**1.20 (0.34; 2.06)**	0.69 (-0.10; 1.48)	0.58 (-0.22; 1.37)	**0.69 (0.07; 1.32)**	AET	.	-0.22 (-1.08; 0.64)	.	.	**-0.92 (-1.45; -0.38)**
**V16**	**-1.50 (-2.54; -0.46)**	-1.16 (-2.48; 0.16)	1.20 (-0.17; 2.57)	-0.85 (-1.82; 0.12)	-0.26 (-1.53; 1.00)	-0.17 (-1.55; 1.22)	-0.37 (-1.62; 0.87)	0.20 (-0.61; 1.01)	-0.81 (-1.77; 0.16)	-0.02 (-1.07; 1.04)	0.75 (-0.27; 1.76)	0.24 (-0.73; 1.21)	0.12 (-0.85; 1.09)	0.24 (-0.59; 1.07)	-0.45 (-1.36; 0.46)	AQET	.	.	.	-0.39 (-1.13; 0.36)
**V17**	**-1.29 (-2.25; -0.33)**	-0.95 (-2.19; 0.29)	**1.41 (0.09; 2.73)**	-0.64 (-1.54; 0.26)	-0.05 (-1.27; 1.16)	0.05 (-1.27; 1.36)	-0.16 (-1.35; 1.02)	0.41 (-0.32; 1.14)	-0.59 (-1.49; 0.30)	0.19 (-0.76; 1.15)	**0.96 (0.01; 1.91)**	0.45 (-0.38; 1.28)	0.33 (-0.54; 1.21)	0.45 (-0.29; 1.20)	-0.24 (-0.95; 0.46)	0.21 (-0.78; 1.20)	MBT	**-0.83 (-1.45; -0.20)**	.	-0.52 (-1.25; 0.21)
**V18**	**-2.12 (-3.27; -0.97)**	**-1.78 (-3.17; -0.39)**	0.58 (-0.88; 2.04)	**-1.46 (-2.56; -0.37)**	-0.88 (-2.25; 0.48)	-0.78 (-2.24; 0.68)	-0.99 (-2.33; 0.35)	-0.42 (-1.38; 0.54)	**-1.42 (-2.52; -0.33)**	-0.63 (-1.78; 0.51)	0.13 (-1.01; 1.27)	-0.38 (-1.42; 0.66)	-0.49 (-1.57; 0.58)	-0.38 (-1.35; 0.60)	**-1.07 (-2.01; -0.13)**	-0.62 (-1.79; 0.56)	**-0.83 (-1.45; -0.20)**	PropT	.	.
**V19**	**-1.73 (-2.91; -0.54)**	-1.39 (-2.82; 0.04)	0.97 (-0.45; 2.39)	**-1.07 (-2.13; -0.01)**	-0.49 (-1.84; 0.86)	-0.39 (-1.88; 1.10)	-0.60 (-1.97; 0.76)	-0.03 (-0.95; 0.90)	-1.03 (-2.15; 0.08)	-0.24 (-1.44; 0.95)	0.52 (-0.56; 1.61)	0.01 (-1.11; 1.13)	-0.10 (-1.22; 1.02)	0.02 (-0.84; 0.88)	-0.68 (-1.74; 0.39)	-0.23 (-1.42; 0.97)	-0.44 (-1.57; 0.70)	0.39 (-0.91; 1.69)	WB-EMS	.
**V20**	**-1.89 (-2.62; -1.16)**	**-1.55 (-2.63; -0.46)**	0.81 (-0.34; 1.96)	**-1.23 (-1.85; -0.62)**	-0.65 (-1.67; 0.37)	-0.55 (-1.72; 0.61)	-0.76 (-1.76; 0.24)	-0.19 (-0.50; 0.12)	**-1.19 (-1.81; -0.58)**	-0.40 (-1.15; 0.34)	0.36 (-0.33; 1.05)	-0.15 (-0.77; 0.47)	-0.26 (-0.88; 0.36)	-0.15 (-0.50; 0.21)	**-0.84 (-1.35; -0.32)**	-0.39 (-1.13; 0.36)	-0.60 (-1.25; 0.06)	0.23 (-0.68; 1.14)	-0.16 (-1.09; 0.77)	RC
	**V1**	**V2**	**V3**	**V4**	**V5**	**V6**	**V7**	**V8**	**V9**	**V10**	**V11**	**V12**	**V13**	**V14**	**V15**	**V16**	**V17**	**V18**	**V19**	**V20**
	Relative effects of NMA (column compared with row)																			

Pairwise (upper right portion) and network (lower left portion) meta-analysis results are presented for mean change (from baseline) in pain outcomes. Effect estimation is presented in standardized mean difference (SMD) with 95% CI. Significant results are marked in bold.

NMA, network meta-analysis; AET, aerobic exercise training; AQET, aquatic exercise therapy; BFR, blood flow restriction; HIRET, high-intensity resistance exercise training; IKET, isokinetic exercise training; IMET, isometric exercise training; LIRET, low-intensity resistance exercise training; MBT, mind-body therapy; MET, multicomponent exercise training; MIRET, medium-intensity resistance exercise training; ND, nutrition and diet; PAM, physical agent modality; PropT, proprioceptive training; RC, regular care; TCM, traditional Chinese medicine; WB-EMS, whole-body electromyostimulation; WBV, whole body vibration.

Supplementary Table S4

Table S4. Summary of compliance & adverse events.

Study (year)	Study arm	Group sample (n)	Withdraw, attrition rate, or drop out (number of patients)			Side effects and complications (number of patients)			Serious adverse event (number of patients)		
			Related to treatment	Unrelated to treatment	Total sum	Related to treatment	Unrelated to treatment	Total sum	Related to treatment	Unrelated to treatment	Total sum
Beavers 2015	Gr 1: MET	150	0	33	33	NR	NR		NR	NR	
	Gr 2: MET + ND	152	0	33	33	NR	NR		NR	NR	
	Gr 3: RC	152	0	34	34	NR	NR		NR	NR	
Bruce-Brand 2012	Gr 1: HIRET	14	1	3	4	NR	NR		NR	NR	
	Gr 2: IMET (NMES)	14	2	2	4	NR	NR		NR	NR	
	Gr 3: RC	13	2	5	7	NR	NR		NR	NR	
Chen 2021	Gr 1: IMET (Active) + TCM	46	0	0	0	0	0	0	0	0	0
	Gr 2: MET	46	0	0	0	0	0	0	0	0	0
Chen 2023	Gr 1: IMET (Active) + TCM	50	0	0	0	NR	NR		NR	NR	
	Gr 2: RC	60	0	0	0	NR	NR		NR	NR	
Choi 2015	Gr 1: IMET (Active) + BioF	20	0	0	0	NR	NR		NR	NR	
	Gr 2: RC	10	0	0	0	NR	NR		NR	NR	
Christensen 2013	Gr 1: MET + ND	64	5	1	6	0	12	12	0	0	0
	Gr 2: RC	128	3	7	10	0	27	27	0	0	0
Cook 2017	Gr 1: MIRET	12	0	1	1	0	1	1	NR	NR	
	Gr 2: LIRET	12	0	0	0	0	0	0	NR	NR	
	Gr 3: LIRET + BFR	12	0	2	2	0	2	2	NR	NR	
de Almeida 2020	Gr 1: AET	22	0	2	2	NR	NR		NR	NR	
	Gr 2: MIRET	22	0	1	1	NR	NR		NR	NR	
	Gr 3: RC	22	0	2	2	NR	NR		NR	NR	
Devrimsel 2019	Gr 1: MET + PAM	30	0	1	1	NR	NR		NR	NR	
	Gr 2: IMET (NMES)	30	0	0	0	NR	NR		NR	NR	
Ferraz 2018	Gr 1: HIRET	16	4	2	6	4	0	4	0	0	0
	Gr 2: LIRET	16	0	4	4	0	1	1	0	0	0
	Gr 3: LIRET + BFR	16	0	4	4	0	2	2	0	0	0
Franz 2022	Gr 1: AET	10	0	0	0	0	0	0	0	0	0
	Gr 2: AET + BFR	10	0	0	0	0	0	0	0	0	0
	Gr 3: RC	10	0	0	0	0	0	0	0	0	0
Gur 2002	Gr 1: IKET	17	0	0	0	0	0	0	0	0	0
	Gr 2: RC	6	0	0	0	0	0	0	0	0	0
Ha 2018	Gr 1: AQET	9	0	0	0	NR	NR		NR	NR	
	Gr 2: RC	8	0	0	0	NR	NR		NR	NR	
Harper 2019	Gr 1: LIRET + BFR	16	3	1	4	6	7	13	1	1	2
	Gr 2: MIRET	19	3	1	4	15	6	21	0	3	3
Ji 2016	Gr 1: MET + TCM	30	0	2	2	NR	NR		NR	NR	
	Gr 2: RC	30	0	1	1	NR	NR		NR	NR	
Jiang 2020	Gr 1: MBT	20	0	9	9	NR	NR		NR	NR	
	Gr 2: RC	20	0	8	8	NR	NR		NR	NR	
Kelmendi 2024	Gr 1: WB-EMS	36	0	5	5	0	0	0	0	0	0
	Gr 2: MET	36	0	1	1	0	0	0	0	0	0
Kim 2021	Gr 1: AQET	20	0	0	0	0	6	6	0	0	0
	Gr 2: RC	23	0	1	1	0	5	5	0	0	0
Kocaman 2008	Gr 1: IMET (Active) + PAM	19	0	0	0	NR	NR		NR	NR	
	Gr 2: IMET (NMES) + PAM	19	0	0	0	NR	NR		NR	NR	
Kuntz 2018	Gr 1: HIRET	11	0	1	1	0	0	0	0	0	0
	Gr 2: MBT	10	0	0	0	0	0	0	0	0	0
	Gr 3: RC	10	0	0	0	0	0	0	0	0	0
Li 2022	Gr 1: MBT	30	0	0	0	0	0	0	0	0	0
	Gr 2: PropT	28	0	0	0	0	0	0	0	0	0
Lin 2022	Gr 1: AET	97	0	0	0	0	16	16	0	0	0
	Gr 2: RC	34	0	0	0	0	8	8	0	0	0
Liu 2019	Gr 1: AET	35	0	8	8	0	16	16	0	0	0
	Gr 2: MBT	70	0	13	13	0	16	16	0	0	0
	Gr 3: RC	35	0	11	11	0	8	8	0	0	0
Lu 2022	Gr 1: MET + TCM	64	0	0	0	0	3	3	0	0	0
	Gr 2: RC	64	0	0	0	0	6	6	0	0	0
Ma 2019	Gr 1: IKET + TCM	92	0	0	0	NR	NR		NR	NR	
	Gr 2: IKET	93	0	0	0	NR	NR		NR	NR	
Mahmoud 2017	Gr 1: IMET (Active)	40	0	8	8	NR	NR		NR	NR	

	Gr 2: RC	20	0	8	8	NR	NR		NR	NR	
Mahmoud 2021	Gr 1: LIRET + BFR	20	0	3	3	NR	NR		NR	NR	
	Gr 2: RC	20	0	2	2	NR	NR		NR	NR	
Malas 2013	Gr 1: LIRET + PAM	22	0	3	3	NR	NR		NR	NR	
	Gr 2: IKET + PAM	22	0	2	2	NR	NR		NR	NR	
	Gr 3: IMET (Active) + PAM	22	0	0	0	NR	NR		NR	NR	
	Gr 4: RC	66	0	5	5	NR	NR		NR	NR	
McLeod 2020	Gr 1: MET + ND	72	0	7	7	NR	NR		NR	NR	
	Gr 2: MET	83	0	9	9	NR	NR		NR	NR	
Melo Mde 2015	Gr 1: IMET (NMES)	15	0	0	0	NR	NR		NR	NR	
	Gr 2: IMET (NMES) + PAM	15	0	1	1	NR	NR		NR	NR	
	Gr 3: RC	15	0	0	0	NR	NR		NR	NR	
Messier 2000	Gr 1: MET + ND	13	0	1	1	NR	NR		NR	NR	
	Gr 2: MET	11	0	2	2	NR	NR		NR	NR	
Messier 2021	Gr 1: HIRET	127	0	19	19	0	1	1	0	0	0
	Gr 2: MIRET	126	0	22	22	0	1	1	0	0	0
	Gr 3: RC	124	0	25	25	0	0	0	0	0	0
Miller 2008	Gr 1: MET + ND	31	0	0	0	NR	NR		NR	NR	
	Gr 2: RC	36	0	0	0	NR	NR		NR	NR	
Mu 2019	Gr 1: IKET + PAM	55	0	0	0	2	0	2	0	0	0
	Gr 2: IKET	54	0	0	0	4	0	4	0	0	0
Nicklas 2004	Gr 1: MET + ND	79	0	15	15	NR	NR		NR	NR	
	Gr 2: MET	79	0	12	12	NR	NR		NR	NR	
	Gr 3: RC	158	0	17	17	NR	NR		NR	NR	
Oldham 1995	Gr 1: IMET (NMES)	22	0	0	0	NR	NR		NR	NR	
	Gr 2: RC	8	0	0	0	NR	NR		NR	NR	
Raeissadat 2018	Gr 1: IMET (Active)	23	0	2	2	NR	NR		NR	NR	
	Gr 2: IMET (Active) + BioF	23	0	3	3	NR	NR		NR	NR	
Samut 2015	Gr 1: AET	14	0	0	0	NR	NR		NR	NR	
	Gr 2: IKET	15	0	2	2	NR	NR		NR	NR	
	Gr 3: RC	13	0	0	0	NR	NR		NR	NR	
Segal 2015	Gr 1: LIRET	24	0	3	3	NR	NR		NR	NR	
	Gr 2: LIRET + BFR	21	0	2	2	NR	NR		NR	NR	
Simao 2012	Gr 1: IMET (Active)	11	0	1	1	NR	NR		NR	NR	
	Gr 2: IMET (Active) + WBV	12	0	1	1	NR	NR		NR	NR	
	Gr 3: RC	12	0	1	1	NR	NR		NR	NR	
Sterzi 2016	Gr 1: IMET (Active) + ND	26	0	3	3	0	0	0	0	0	0
	Gr 2: IMET (Active)	27	0	0	0	0	0	0	0	0	0
Tok 2011	Gr 1: IMET (Active) + PAM	20	0	0	0	0	0	0	0	0	0
	Gr 2: IMET (NMES) + PAM	20	0	0	0	0	0	0	0	0	0
Varzaityte 2020	Gr 1: MET	30	0	0	0	0	0	0	0	0	0
	Gr 2: AQET	30	0	0	0	0	0	0	0	0	0
Vassao 2021	Gr 1: MIRET + PAM	14	1	0	1	NR	NR		NR	NR	
	Gr 2: MIRET	14	1	0	1	NR	NR		NR	NR	
	Gr 3: RC	14	4	0	4	NR	NR		NR	NR	
Walls 2010	Gr 1: IMET (Active)	5	0	0	0	0	0	0	0	0	0
	Gr 2: IMET (NMES)	9	0	0	0	0	0	0	0	0	0
Walrabenstein 2023	Gr 1: MET + ND	33	0	1	1	0	3	3	0	0	0
	Gr 2: RC	33	0	1	1	0	7	7	0	0	0
Wang 2016	Gr 1: MET + TCM	30	0	0	0	0	0	0	0	0	0
	Gr 2: RC	30	0	0	0	0	0	0	0	0	0
Wang 2017	Gr 1: MET + TCM	45	0	0	0	0	0	0	0	0	0
	Gr 2: RC	45	0	0	0	0	0	0	0	0	0
Wang 2021	Gr 1: IMET (Active)	75	0	0	0	NR	NR		NR	NR	
	Gr 2: MET	75	0	0	0	NR	NR		NR	NR	
Wyatt 2001	Gr 1: AQET	23	0	2	2	0	2	2	0	0	0
	Gr 2: MET	23	0	2	2	0	2	2	0	0	0
Yang 2023	Gr 1: MBT	42	0	0	0	0	0	0	0	0	0
	Gr 2: PropT	42	1	0	1	0	0	0	0	0	0
Yin 2021	Gr 1: IKET + PAM	35	0	0	0	NR	NR		NR	NR	
	Gr 2: RC	35	0	0	0	NR	NR		NR	NR	

AET, aerobic exercise training; AQET, aquatic exercise therapy; BFR, blood flow restriction; BioF, biofeedback; HIRET, high-intensity resistance exercise training; IKET, isokinetic exercise training; IMET, isometric exercise training; LIRET, low-intensity resistance exercise training; MBT, mind-body therapy; MET, multicomponent exercise training; MIRET, medium-intensity resistance exercise training; ND, nutrition and diet; NMES, neuromuscular electrical stimulation; PAM, physical agent modality; PropT, proprioceptive training; RC, regular care; TCM, traditional Chinese medicine; WB-EMS, whole-body electromyostimulation; WBV, whole body vibration; NR, not reported.

Supplementary Figure S1

	Random sequence generation (selection bias)	Allocation concealment (selection bias)	Similarity at the baseline (selection bias)	Blinding of participants and personnel (performance bias)	Therapist blinding (performance bias)	Blinding of outcome assessment (detection bias)	Adequate follow-up participants (attrition bias)	Intention-to-treat analysis (attrition bias)	Between-group difference (attrition bias)	Point and variability measures	Selective reporting (reporting bias)	Author conflict of interest disclosures (unconscious bias)	Other bias	Overall
Beavers 2015	?	?	+	−	?	+	−	−	+	+	+	+	+	−
Bruce-Brand 2012	−	−	+	−	−	+	−	−	+	+	?	+	+	−
Chen 2021	?	?	+	−	?	?	+	+	+	+	+	?	?	?
Chen 2023	?	?	+	?	?	?	+	+	+	+	+	?	?	?
Choi 2015	?	?	+	−	?	+	?	?	+	+	+	?	?	?
Christensen 2013	+	+	+	−	?	+	+	+	+	+	+	+	+	?
Cook 2017	?	?	+	?	?	+	+	+	+	+	+	+	+	+
de Almeida 2020	+	+	+	−	−	+	+	−	+	+	?	+	?	−
Devrimsel 2019	+	+	+	?	?	+	+	+	+	+	+	+	+	+
Ferraz 2018	?	?	+	?	?	+	−	+	+	+	+	+	?	?
Franz 2022	?	?	+	+	?	+	+	+	+	+	+	?	?	+
Gur 2002	?	?	+	?	?	?	+	+	+	+	+	+	?	+
Ha 2018	?	?	+	−	?	?	+	+	+	+	+	+	+	?
Harper 2019	+	+	+	?	?	+	−	+	+	+	+	?	?	?
Ji 2016	+	?	+	−	?	?	+	−	+	+	+	?	?	?
Jiang 2020	?	?	+	−	?	+	−	−	+	+	+	?	?	−
Kelmendi 2024	+	+	+	−	+	+	+	+	+	+	+	+	?	?
Kim 2021	+	?	+	−	−	−	+	+	+	+	+	+	?	−
Kocaman 2008	?	?	+	−	−	+	?	?	+	+	−	?	?	−
Kuntz 2018	+	+	+	+	+	+	+	−	+	+	+	+	?	?
Li 2022	+	?	+	−	−	−	+	+	+	+	+	?	?	−
Lin 2022	+	?	+	−	−	?	+	+	+	+	+	+	?	?
Liu 2019	+	?	+	−	−	−	+	+	+	+	+	+	?	−
Lu 2022	+	?	+	+	?	?	+	+	+	+	+	?	−	?
Ma 2019	?	?	+	?	?	?	+	+	+	+	+	?	?	?
Mahmoud 2017	?	?	+	−	?	+	?	+	+	+	+	+	?	?
Mahmoud 2021	?	+	+	?	?	+	+	−	+	+	+	+	?	?
Malas 2013	?	?	+	?	?	+	+	−	+	+	?	+	+	?
McLeod 2020	+	+	+	−	?	−	+	+	+	+	+	+	+	?
Melo Mde 2015	+	+	+	−	−	+	+	−	+	+	+	+	+	−
Messier 2000	?	?	+	−	−	+	+	−	+	+	+	?	?	−
Messier 2021	+	?	+	−	?	+	+	?	+	+	+	+	?	?
Miller 2008	?	?	+	?	?	?	+	+	+	+	+	+	−	?
Mu 2019	+	?	+	?	?	?	+	+	+	+	+	?	?	?
Nicklas 2004	+	+	+	−	−	+	+	−	+	+	+	?	?	−
Oldham 1995	?	?	+	+	−	+	+	?	+	+	+	?	−	?
Raeissadat 2018	?	?	+	+	−	+	+	−	+	+	+	+	?	?
Samut 2015	?	?	+	−	−	?	+	+	+	+	+	+	?	?
Segal 2015	+	+	+	+	−	+	+	−	+	+	+	+	?	?
Simao 2012	+	+	+	−	−	+	+	−	+	+	+	+	+	−
Sterzi 2016	+	+	+	+	+	+	+	−	+	+	+	+	+	?
Tok 2011	?	?	+	−	−	?	+	+	+	+	+	?	?	?
Varzaityte 2020	+	?	+	?	+	+	+	+	+	+	+	+	?	+
Vassao 2021	+	+	+	+	?	+	+	−	+	+	+	+	?	?
Walls 2010	+	?	+	−	?	+	−	−	+	+	+	+	+	−
Walrabenstein 2023	+	+	+	−	?	+	+	−	+	+	+	+	+	?
Wang 2016	+	?	+	−	?	?	+	+	+	+	+	?	?	?
Wang 2017	?	?	+	−	?	?	+	+	+	+	+	?	?	?
Wang 2021	+	?	+	−	?	?	+	+	+	+	+	?	?	?
Wyatt 2001	?	?	+	−	?	+	+	+	+	+	+	?	?	?
Yang 2023	?	?	+	−	?	+	+	−	+	+	+	?	?	?
Yin 2021	+	?	+	−	?	?	+	+	+	+	+	?	?	?

Supplementary Figure S2

Comparison	Number of Studies	Direct Evidence	I2	SMD	95%CI
**AET:AET + BFR**					
Direct estimate	1	0.85		−0.27	[−1.51; 0.97]
Indirect estimate				−0.19	[−3.11; 2.74]
Network estimate				−0.25	[−1.40; 0.89]
**AET:MIRET**					
Direct estimate	1	0.61		−0.47	[−1.54; 0.60]
Indirect estimate				−0.40	[−1.75; 0.95]
Network estimate				−0.44	[−1.28; 0.40]
**AET:RC**					
Direct estimate	2	0.89	0%	0.28	[−0.53; 1.09]
Indirect estimate				0.09	[−2.21; 2.39]
Network estimate				0.26	[−0.51; 1.02]
**AET + BFR:RC**					
Direct estimate	1	0.84		0.50	[−0.74; 1.75]
Indirect estimate				0.58	[−2.32; 3.48]
Network estimate				0.51	[−0.63; 1.66]
**HIRET:IMET (NMES)**					
Direct estimate	1	0.37		−0.07	[−1.30; 1.17]
Indirect estimate				−0.15	[−1.11; 0.80]
Network estimate				−0.12	[−0.88; 0.63]
**HIRET:LIRET**					
Direct estimate	1	0.65		0.36	[−0.75; 1.48]
Indirect estimate				0.57	[−0.95; 2.10]
Network estimate				0.44	[−0.47; 1.34]
**HIRET:LIRET + BFR**					
Direct estimate	1	0.43		0.06	[−1.05; 1.18]
Indirect estimate				−1.20	[−2.17; −0.23]
Network estimate				−0.66	[−1.39; 0.07]
**HIRET:MIRET**					
Direct estimate	1	0.57		0.01	[−0.92; 0.95]
Indirect estimate				−0.19	[−1.27; 0.88]
Network estimate				−0.07	[−0.78; 0.63]
**HIRET:RC**					
Direct estimate	2	0.65	0%	0.23	[−0.53; 1.00]
Indirect estimate				1.36	[0.31; 2.41]
Network estimate				0.63	[0.01; 1.24]
**IKET + PAM:IMET (Active) + PAM**					
Direct estimate	1	0.88		−0.08	[−1.15; 0.98]
Indirect estimate				−1.29	[−4.16; 1.57]
Network estimate				−0.23	[−1.23; 0.77]
**IKET + PAM:RC**					
Direct estimate	1	0.91		0.09	[−0.92; 1.10]
Indirect estimate				1.45	[−1.84; 4.74]
Network estimate				0.21	[−0.76; 1.17]
**IMET (Active):IMET (Active) + BioF**					
Direct estimate	1	0.66		−0.40	[−1.47; 0.67]
Indirect estimate				−0.05	[−1.53; 1.44]
Network estimate				−0.28	[−1.15; 0.59]
**IMET (Active):IMET (NMES)**					
Direct estimate	1	0.36		−0.90	[−2.34; 0.54]
Indirect estimate				0.23	[−0.85; 1.31]
Network estimate				−0.18	[−1.04; 0.69]
**IMET (Active):RC**					
Direct estimate	1	0.49		1.15	[0.02; 2.27]
Indirect estimate				0.01	[−1.10; 1.12]
Network estimate				0.57	[−0.22; 1.36]
**IMET (Active) + BioF:RC**					
Direct estimate	1	0.59		0.70	[−0.47; 1.88]
Indirect estimate				1.06	[−0.35; 2.46]
Network estimate				0.85	[−0.05; 1.75]
**IMET (Active) + PAM:IMET (NMES) + PAM**					
Direct estimate	2	0.70	22%	−0.27	[−1.09; 0.56]
Indirect estimate				−0.86	[−2.12; 0.39]
Network estimate				−0.45	[−1.14; 0.24]
**IMET (Active) + PAM:LIRET + PAM**					
Direct estimate	1	0.88		0.17	[−0.90; 1.24]
Indirect estimate				1.39	[−1.50; 4.28]
Network estimate				0.31	[−0.69; 1.32]
**IMET (Active) + PAM:RC**					
Direct estimate	1	0.56		0.17	[−0.83; 1.17]
Indirect estimate				0.77	[−0.35; 1.89]
Network estimate				0.44	[−0.31; 1.18]
**IMET (NMES):IMET (NMES) + PAM**					
Direct estimate	2	0.75	0%	−0.14	[−0.94; 0.67]
Indirect estimate				−0.12	[−1.52; 1.28]
Network estimate				−0.13	[−0.83; 0.56]
**IMET (NMES):RC**					
Direct estimate	4	0.81	31%	0.67	[0.07; 1.27]
Indirect estimate				1.08	[−0.15; 2.32]
Network estimate				0.75	[0.21; 1.29]
**IMET (NMES) + PAM:RC**					
Direct estimate	2	0.64	0%	1.15	[0.33; 1.97]
Indirect estimate				0.41	[−0.68; 1.50]
Network estimate				0.88	[0.22; 1.54]
**LIRET:LIRET + BFR**					
Direct estimate	2	0.93	86%	−0.98	[−1.78; −0.19]
Indirect estimate				−2.49	[−5.31; 0.34]
Network estimate				−1.09	[−1.86; −0.33]
**LIRET + BFR:MIRET**					
Direct estimate	1	0.42		0.18	[−1.01; 1.36]
Indirect estimate				0.88	[−0.13; 1.89]
Network estimate				0.58	[−0.19; 1.35]
**LIRET + BFR:RC**					
Direct estimate	2	0.64	0%	1.72	[0.87; 2.58]
Indirect estimate				0.49	[−0.66; 1.64]
Network estimate				1.28	[0.60; 1.97]
**LIRET + PAM:RC**					
Direct estimate	1	0.91		0.00	[−1.01; 1.02]
Indirect estimate				1.38	[−1.94; 4.71]
Network estimate				0.12	[−0.85; 1.09]
**MIRET:RC**					
Direct estimate	3	0.88	79%	0.62	[0.01; 1.23]
Indirect estimate				1.32	[−0.37; 3.01]
Network estimate				0.70	[0.13; 1.27]

Random Effects Model

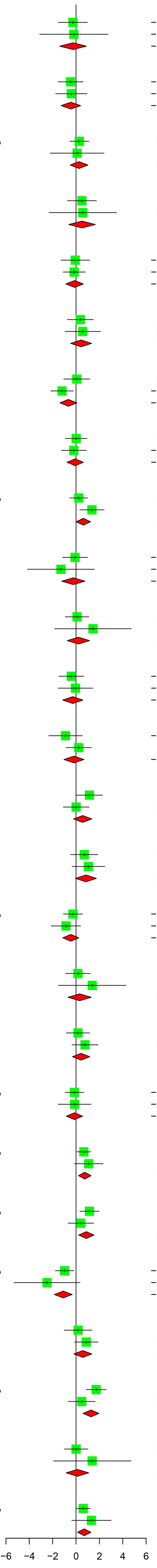

Supplementary Figure S3

Comparison	Number of Studies	Direct Evidence	I2	SMD	95%CI
**AET vs IKET**					
Direct estimate	1	0.61		0.27	[−0.76; 1.30]
Indirect estimate				−1.52	[−2.80; −0.24]
Network estimate				−0.43	[−1.24; 0.37]
**AET vs MBT**					
Direct estimate	1	0.67		−0.22	[−1.08; 0.64]
Indirect estimate				−0.29	[−1.52; 0.95]
Network estimate				−0.24	[−0.95; 0.46]
**AET vs RC**					
Direct estimate	3	0.93	72%	−0.92	[−1.45; −0.38]
Indirect estimate				0.25	[−1.76; 2.26]
Network estimate				−0.84	[−1.35; −0.32]
**HIRET vs MBT**					
Direct estimate	1	0.41		0.67	[−0.63; 1.97]
Indirect estimate				0.30	[−0.78; 1.38]
Network estimate				0.45	[−0.38; 1.28]
**HIRET vs MIRET**					
Direct estimate	1	0.81		−0.04	[−0.82; 0.74]
Indirect estimate				0.75	[−0.83; 2.33]
Network estimate				0.12	[−0.58; 0.81]
**HIRET vs RC**					
Direct estimate	2	0.89	0%	−0.10	[−0.76; 0.57]
Indirect estimate				−0.57	[−2.42; 1.29]
Network estimate				−0.15	[−0.77; 0.47]
**IKET vs IKET + PAM**					
Direct estimate	1	0.69		1.98	[1.12; 2.83]
Indirect estimate				0.36	[−0.93; 1.65]
Network estimate				1.48	[0.77; 2.20]
**IKET vs RC**					
Direct estimate	1	0.51		−0.77	[−1.82; 0.28]
Indirect estimate				−0.03	[−1.10; 1.03]
Network estimate				−0.40	[−1.15; 0.34]
**IKET + PAM vs RC**					
Direct estimate	1	0.67		−1.35	[−2.25; −0.46]
Indirect estimate				−2.97	[−4.24; −1.70]
Network estimate				−1.89	[−2.62; −1.16]
**IMET (Active) vs IMET (Active) + WBV**					
Direct estimate	1	0.85		0.54	[−0.56; 1.64]
Indirect estimate				3.75	[1.09; 6.41]
Network estimate				1.01	[−0.01; 2.03]
**IMET (Active) vs MET**					
Direct estimate	1	0.68		1.01	[0.21; 1.81]
Indirect estimate				−0.56	[−1.72; 0.60]
Network estimate				0.51	[−0.15; 1.17]
**IMET (Active) vs RC**					
Direct estimate	1	0.39		−0.60	[−1.70; 0.50]
Indirect estimate				0.97	[0.09; 1.86]
Network estimate				0.36	[−0.33; 1.05]
**IMET (Active) + TCM vs MET**					
Direct estimate	1	0.54		−0.71	[−1.54; 0.13]
Indirect estimate				−1.54	[−2.46; −0.63]
Network estimate				−1.09	[−1.71; −0.47]
**IMET (Active) + TCM vs RC**					
Direct estimate	1	0.55		−1.61	[−2.45; −0.78]
Indirect estimate				−0.78	[−1.69; 0.14]
Network estimate				−1.23	[−1.85; −0.62]
**IMET (Active) + WBV vs RC**					
Direct estimate	1	0.84		−1.14	[−2.25; −0.03]
Indirect estimate				2.00	[−0.59; 4.58]
Network estimate				−0.65	[−1.67; 0.37]
**MBT vs RC**					
Direct estimate	2	0.81	0%	−0.52	[−1.25; 0.21]
Indirect estimate				−0.94	[−2.44; 0.57]
Network estimate				−0.60	[−1.25; 0.06]
**MET vs MET + ND**					
Direct estimate	5	0.86	0%	0.13	[−0.24; 0.50]
Indirect estimate				−0.49	[−1.41; 0.43]
Network estimate				0.04	[−0.30; 0.38]
**MET vs RC**					
Direct estimate	3	0.64	51%	0.13	[−0.32; 0.57]
Indirect estimate				−0.63	[−1.23; −0.04]
Network estimate				−0.15	[−0.50; 0.21]
**MET + ND vs RC**					
Direct estimate	6	0.91	23%	−0.12	[−0.45; 0.21]
Indirect estimate				−0.90	[−1.96; 0.16]
Network estimate				−0.19	[−0.50; 0.12]
**MIRET vs MIRET + PAM**					
Direct estimate	1	0.85		0.28	[−0.78; 1.34]
Indirect estimate				1.76	[−0.77; 4.29]
Network estimate				0.50	[−0.48; 1.47]
**MIRET vs RC**					
Direct estimate	2	0.94	59%	−0.29	[−0.92; 0.35]
Indirect estimate				0.09	[−2.52; 2.71]
Network estimate				−0.26	[−0.88; 0.36]
**MIRET + PAM vs RC**					
Direct estimate	1	0.79		−1.04	[−2.16; 0.08]
Indirect estimate				0.28	[−1.90; 2.47]
Network estimate				−0.76	[−1.76; 0.24]

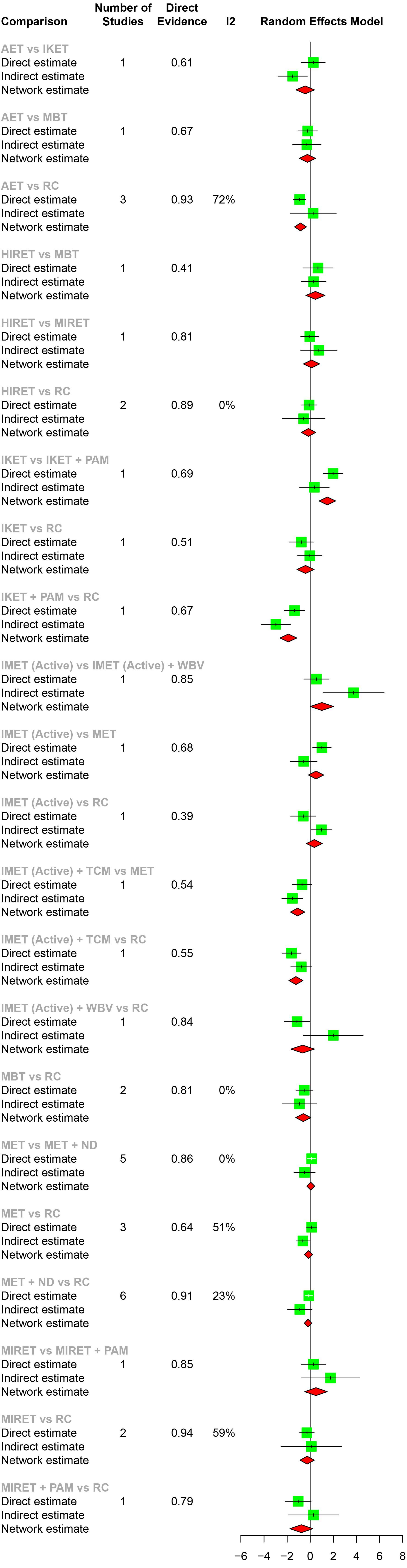

Supplement: Supplementary file 1 [file biomedicines-12-01524-s001.zip › biomedicines-3054226-supplementary.pdf]
